# Gastrointestinal Stromal Tumor Recurrence Presenting as a Small Bowel Obstruction: A Case Report

**DOI:** 10.7759/cureus.33806

**Published:** 2023-01-15

**Authors:** Dana A Neama, Sarah J Al Araibi, Amer E Alderazi, Sayed Ali I Almahari, Abdulrahman M Alawadhi

**Affiliations:** 1 General Surgery, Salmaniya Medical Complex, Manama, BHR; 2 General and Bariatric Surgery, Salmaniya Medical Complex, Manama, BHR; 3 Pathology and Laboratory Medicine, Salmaniya Medical Complex, Manama, BHR

**Keywords:** tyrosine kinase inhibitor, tki, small bowel obstruction, abdominal mass, gist, gastrointestinal stromal tumor

## Abstract

Gastrointestinal stromal tumors (GISTs) are the most common mesenchymal tumors of the alimentary tract in adults. The most common site is the stomach, followed by the small intestine. The clinical presentation varies from an incidental finding in asymptomatic patients to a large palpable mass causing complications such as bowel obstruction or viscus perforation. The best imaging modality is a CT scan of the abdomen. Treatment is determined by the size and location of the GISTs. Surgical intervention is considered for resectable tumors, while tyrosine kinase inhibitor therapy is considered for irresectable, metastatic, or recurrent GISTs. In this case report, we present a 30-year-old female who is a known case of gastric GIST and liver metastases. She presented to the emergency department with intestinal obstruction secondary to a recurrent GIST abdominal mass and underwent emergency laparotomy for mass resection. Following surgery, the patient developed aspiration pneumonia, which was treated with proper antibiotics. She was discharged in stable clinical condition with a recommendation to start alternative tyrosine kinase therapy. GISTs are difficult to diagnose preoperatively, as most patients are asymptomatic, and they may present with complications, as in our case, a small bowel obstruction. A proper imaging modality will guide the physician toward the diagnosis, but the final diagnosis will be achieved by biopsy. The diagnosis may be challenging, as small bowel obstruction has many causes, although GISTs should be kept in mind as one of the deferential diagnoses.

## Introduction

Gastrointestinal stromal tumors (GISTs) constitute a rare entity among gastrointestinal (GI) tumors, accounting for less than 1% of GI tumors. They are a type of mesenchymal tumor that arises from interstitial cells of Cajal (ICC) in the GI tract. The most common site is the stomach (50-60%), followed by the small intestine (20-30%), colon and rectum (5%), and the esophagus (5%) [1,2]. Extra-GI sites, such as pancreatic, omental, mesenteric, and retroperitoneal GISTs, have been reported but are very rare [3].

A third of the cases have a malignant course. The clinical condition, the site of the tumor, and the immunohistochemical pattern all play a role in the diagnosis of GISTs. The majority of GISTs are c-KIT positive, with less than 5% being c-KIT negative, particularly those with platelet-derived growth factor receptor alpha (PDGFRA) mutations. The standard modality in the treatment of a GIST is surgical resection if the tumor is resectable without metastasis. However, a tyrosine kinase inhibitor (TKI), such as imatinib, is the main modality of treatment if the tumor is unresectable, metastatic, or recurrent [4-6]. The recurrence rate after surgical resection is common; it depends mostly on three factors: the location, mitotic activity, and size of the tumor [7,8]. In this article, we present a case of a female patient with a recurrent GIST in the large intestine, causing a huge mass effect leading to small bowel obstruction.

## Case presentation

A 30-year-old Indian female had a medical history of gastric GIST with liver metastasis. She underwent surgical resection, which included a total gastrectomy with Roux-en-Y reconstruction, liver lobe resection, and a splenectomy, followed by adjuvant therapy (TKI - imatinib). The patient was lost to follow-up after two years of receiving adjuvant therapy. She was scheduled for several follow-up visits with the oncology department, but she failed to attend any of them.

The patient presented to the emergency department with a complaint of severe generalized abdominal pain, which felt more on the left side and had been present for two days but had become more severe at the time of presentation. It was associated with nausea and multiple vomiting episodes, but no history of constipation was reported. The patient was asked about her previous medical and imaging reports regarding GISTs, and she stated that she did not have them.

On physical examination, she was hemodynamically stable but in severe pain. A clinical examination of the abdomen revealed a distended abdomen and a previous midline laparotomy scar. A well-defined mass was felt in the right hypochondrium with mild generalized tenderness, and no peritoneal signs were observed. Otherwise, her systemic examination was unremarkable.

**Figure 1:**
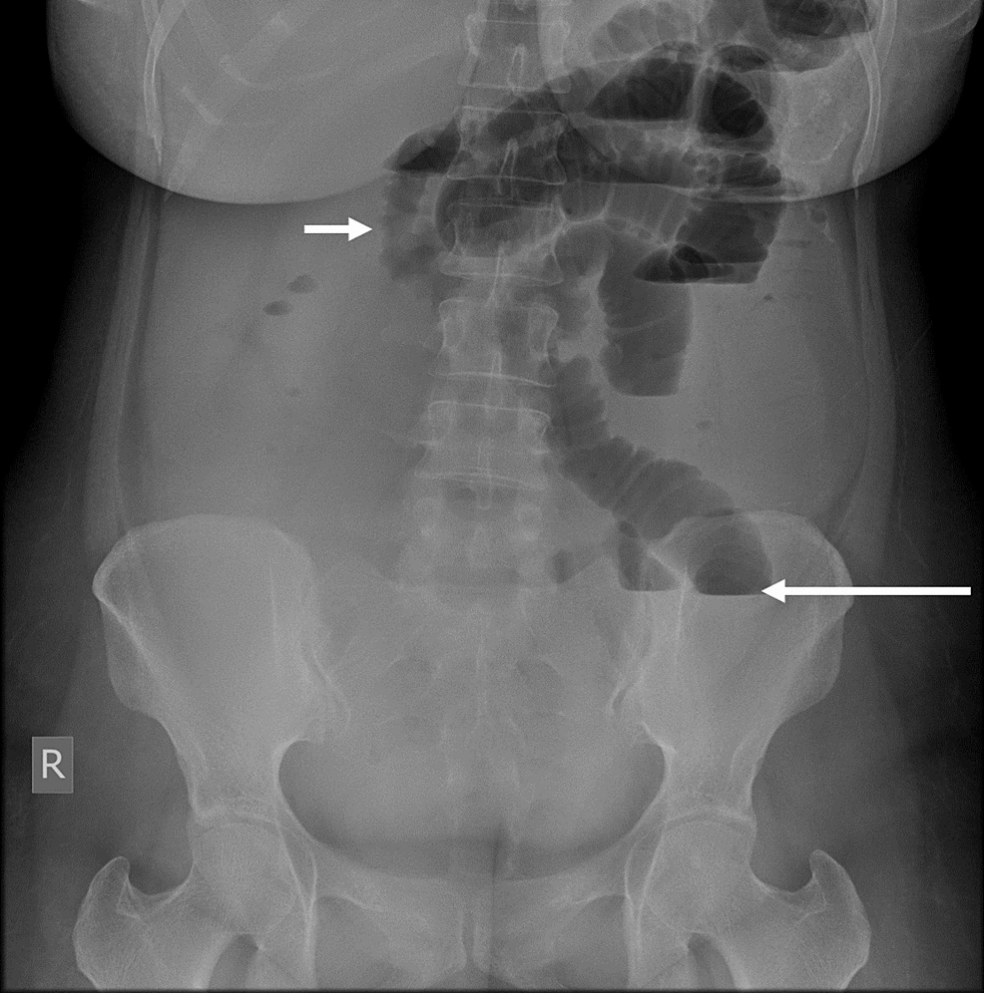
Plain film of the abdomen The long arrow shows multiple air-fluid levels, and the short arrow shows distended small bowel loops.

**Figure 2:**
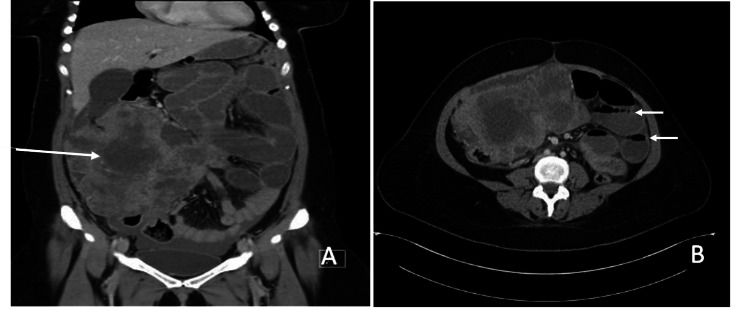
CT scan of the abdomen (A) Coronal view: The long arrow shows a large irregular necrotic mass (most probably a gastrointestinal stromal tumor) measuring about 17.2 x 10.7 x 18.2 cm (mediolateral x anteroposterior x craniocaudal). (B) Transverse view: The short arrows show multiple air-fluid levels at small intestine loops suggestive of small bowel obstruction.

Blood work included complete blood count, amylase, and C-reactive protein (Table 1). An initial abdominal X-ray was done and showed an air-fluid level with small bowel dilatation and shifting of small bowel loops to the left side of the abdomen (Figure 1). An abdominal computed tomography (CT) scan was done and revealed a large right hemi-abdominal irregular necrotic mass (most probably a GIST) measuring about 17.2 x 10.7 x 18.2 cm (mediolateral x anteroposterior x craniocaudal), extending from the subhepatic region to the pelvis. The mass was displacing the adjacent small bowel loops to the left hemi-abdomen, causing upstream dilatation of the proximal ileal and jejunal loops, which appear dilated about 5 cm, with some areas showing air-fluid levels. Moreover, the CT scan findings were suggestive of small bowel obstruction with a transition zone seen at the proximal/mid-ileal loops, most likely secondary to the mass. No pneumoperitoneum could be noted (Figure 2).

**Table 1:** Blood laboratory results

Parameter	Admission laboratory values	Postoperative laboratory values	Discharge laboratory values	Reference range of Salmaniya Medical Complex Laboratory
White blood count (10^9^/L)	20.10	18.63	11.94	3.6-9.6
Hemoglobin (g/dL)	11.3	10.1	12.2	5.2-12.0
Platelets (10^9^/L)	526	393	299	150.0-400.0
Amylase (U/L)	195			30-118
C-reactive protein (mg/L)	262	231	44.3	0-3

The patient underwent emergency laparotomy and right hemicolectomy with primary anastomosis between the distal ileum and the distal 2/3 of the transverse colon. The intraoperative findings revealed a large, malignant mass (2.2 kg), most likely arising from the caecum and extending to involve the proximal third of the transverse colon. The mass was ulcerating, necrotic, and hypervascular. Besides that, the mass was adherent to the right lateral wall and to the small bowel medially, displacing it to the left side. Following surgery, the patient was shifted to the high-dependency unit for observation.

**Figure 3:**
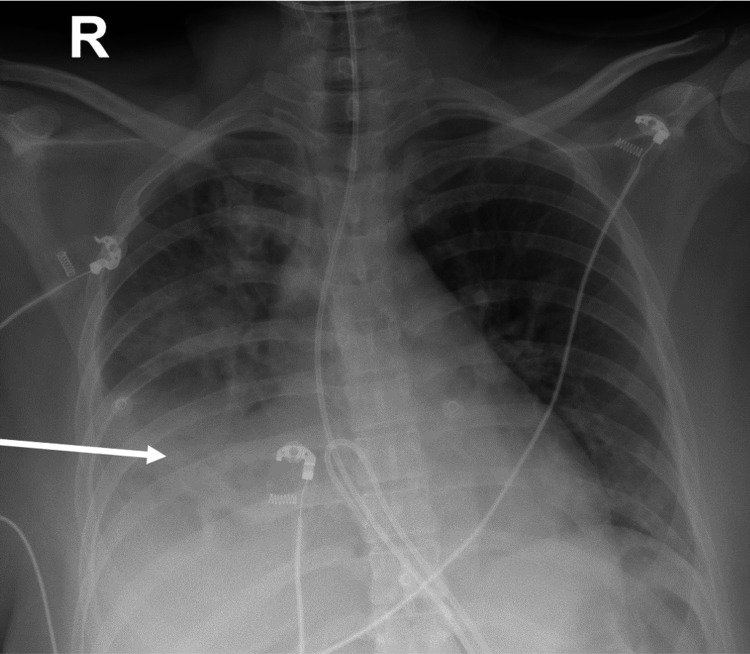
Plain film of the chest The arrow shows consolidation in the lower lobe of the right lung suggesting aspiration pneumonia.

On day one postoperation, the patient developed a fever with episodes of desaturation reaching oxygen saturation (SPO2) of 85%. Upon that, the patient was kept on a face mask with 6 liters of oxygen, which reached SPO2 around 96%. Moreover, further laboratory tests were requested (Table 1). A portable chest X-ray was done and showed consolidation in the right lower lobe, suggesting aspiration pneumonia (Figure 3). A CT pulmonary angiogram was scheduled for her to rule out the possibility of a pulmonary embolism (PE). Although it did not show any conclusive evidence of a PE, the CT scan confirmed the diagnosis of aspiration pneumonia. A chest review was requested regarding pneumonia, and they advised a course of IV antibiotics (meropenem and vancomycin), which she started. The patient was transferred to the general ward after four days since she was starting to feel better and her SPO2 was reaching 98% on room air. The patient was discharged in stable condition with a follow-up visit.

**Figure 4:**
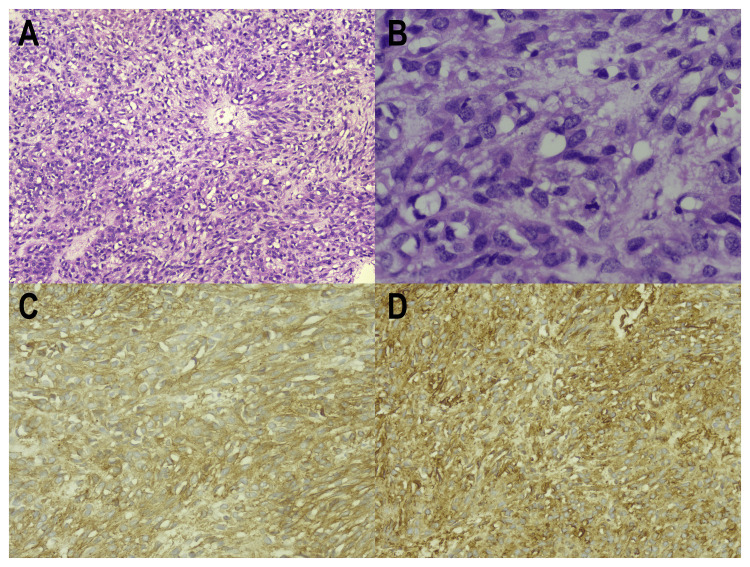
Histopathological slides of the specimen (A, B) Hematoxylin and eosin (H&E) stain with magnifications of x20 and x40, respectively, shows a tumor composed predominantly of spindle cells with intracytoplasmic vacuoles, moderate nuclear pleomorphism, and increased mitotic activity reaching up to 39/5 mm2. (C) Section with magnifications of x20 shows CD34 strong membranous positivity. (D) Section with magnifications of x20 shows DOG1 strong cytoplasmic and membranous positivity.

She returned to the clinic two weeks later to know the result of the histopathology of the excised mass, which revealed features of a GIST: grade 2 (high grade), T4 N0 M0 staging, and tumor type (GIST, spindle cell type, necrosis present 25%). In addition, all the resection margins of the mass were free of tumor cells (Figure 4). As the patient had taken the maximal dose of imatinib in the past, she was advised to begin adjuvant therapy (an alternate TKI, sunitinib) and undergo further investigations, but she preferred to continue her treatment in India.

## Discussion

GISTs are the most common mesenchymal tumors of the alimentary tract [3]. The estimated annual incidence ranges from 11 to 15 cases per million people [1]. Sporadic GISTs occur at any age, with a slight male predominance [9]. Succinate dehydrogenase (SDH) mutant GISTs are well-known to affect children and adolescents. Most of these cases arise in the stomach, with a slight female predominance [10].

GISTs are usually diagnosed as solitary lesions, which can range in size from less than 1 cm to as large as 30 cm in diameter [11]. However, most of the lesions are diagnosed incidentally, with 18% of patients being asymptomatic. The majority of patients present with vague symptoms at the time of diagnosis [12]. The presentation of GISTs highly depends on the site and the size of the tumor, so it can be discovered incidentally in asymptomatic patients with a small tumor or it can present as a life-threatening emergency in the case of viscus perforation. The most common complaint is vague abdominal pain or discomfort [12]. Other complaints include early satiety, abdominal fullness, and dysphagia. If it is associated with blood loss in the stomach or small intestinal GISTs, patients may present with fatigue, malaise, or dyspnea. Furthermore, palpable abdominal mass is a very rare presentation in large GISTs [12].

Morphologically, GISTs can be classified into three main groups: spindle cells (70%), epithelioid (20%), and mixed spindle and epithelioid cell types (10%) [12]. Immunohistochemistry has been found helpful to distinguish between GISTs and other gastrointestinal mesenchymal lesions such as leiomyoma and schwannoma [5]. GISTs are derived from CD34 stem cells, which differentiate into ICC. CD34 testing is positive in 60% to 70% of cases, but it is not specific for GISTs. It mostly occurs due to the activation of oncogenic mutations in the receptor tyrosine kinase gene c-KIT [7]. Thereby, c-KIT-positive (CD117) GISTs account for 95%, while KIT-negative GISTs account for only 5%. It has been evident that c-KIT-negative GISTs are caused mostly by oncogenic mutations in PDGFRA, which help to distinguish between c-KIT-negative GISTs and other GI mesenchymal tumors by genetic testing [7]. DOG1 (gastrointestinal stromal tumor 1) is a GIST-specific gene that is found in roughly 50% of c-KIT-negative GISTs [1]. In our case, c-KIT was negative, mostly due to the use of imatinib for the treatment of the previous GIST [13].

More than 80% of GISTs harbor a gain-of-function of KIT or PDGFRA oncogenes [14,15]. Most of the cases that have wild-type mutations for KIT or PDGFRA have mutations in SDH subunit genes (5-10%) [1].

The diagnosis of GISTs is very challenging. It may involve a routine blood workup to assess the patient's current complaint, but there are no specific blood markers for GISTs. Imaging tests include CT scan, MRI, endoscopy with or without endoscopic ultrasound, and biopsy. However, biopsy and immunohistochemical staining are the only specific diagnostic modalities for GISTs [5].

The preferred treatment option is surgical resection for tumors less than 2 cm without metastasis, which offers a chance for complete remission [5]. On the other hand, TKIs are recommended for irresectable GISTs, metastasis, and recurrent GISTs [5]. Resectable GISTs without metastases will be treated surgically, with the goal of achieving complete resection [5]. Surgical resection may be accomplished by both open and laparoscopic techniques, with the literature suggesting that the laparoscopic approach may be equal to the open approach in gastric GISTs [5]. TKI (imatinib) for three years post-surgical resection in high-risk patients is recommended [5]. Adjuvant imatinib therapy has been shown to improve overall survival and recurrence-free survival rates [5]. Complications like GI hemorrhage, obstruction, and bowel perforation have been noticed more with larger tumor sizes [12].

Lymph node resection is not advised as GISTs usually metastasize to the liver and peritoneum later in the course of the disease [5]. Lymph node involvement is rare, occurring in only 0-8% of patients. However, if lymph nodes are involved, resection must be done [5]. It is worth mentioning that abdominal dissemination increased dramatically in cases of intraoperative tumor rupture [1,8]. In the five-year survival rate, recurrence has been observed in more than 50% of patients even after complete resection of primary GISTs [5].

Important prognostic factors are anatomic site, tumor size, and mitotic activity, which are the basis for the prediction of the risk of aggressive biologic behavior. However, it has been noticed that tumor size is an independent prognostic factor even in tumors with a low mitotic rate [7]. Poor prognostic factors include mitotic rate greater than five mitoses per 50 high-power fields (HPFs) and size greater than 5 cm and 10 cm, which pose a moderate and high malignant potential, respectively. Also, if located outside the stomach, especially in a small bowl, GISTs tend to be more aggressive than those in the stomach [9,12].

## Conclusions

Despite the fact that gastrointestinal tumors (GISTs) are the most common mesenchymal tumors arising from the GI tract, preoperative diagnosis might be difficult. Thus, taking a proper history and choosing the proper imaging will guide the physician to a definitive diagnosis. Always keep in mind that GISTs might be the cause of intestinal obstruction. Many approaches to GIST management exist, including the recently introduced TKI used as adjuvant therapy in addition to surgical resection of the mass with a clear margin or for irresectable, recurrent GISTs. Always consider the risk of recurrence in patients with a history of GISTs and ensure proper follow-up with the use of proper chemotherapy and further imaging to rule out possible recurrence and metastasis.

## References

[1] Sbaraglia M, Businello G, Bellan E, Fassan M, Dei Tos AP (2021). Mesenchymal tumours of the gastrointestinal tract. Pathologica.

[2] Rossi S, Miceli R, Messerini L (2011). Natural history of imatinib-naive GISTs: a retrospective analysis of 929 cases with long-term follow-up and development of a survival nomogram based on mitotic index and size as continuous variables. Am J Surg Pathol.

[3] Elgeidie A, El-Magd EA, El-Maaty SR, El-Hawary AK (2016). Pancreatic gastrointestinal stromal tumor: a case report. Int J Surg Case Rep.

[4] Medeiros F, Corless CL, Duensing A (2004). KIT-negative gastrointestinal stromal tumors: proof of concept and therapeutic implications. Am J Surg Pathol.

[5] Akahoshi K, Oya M, Koga T, Shiratsuchi Y (2018). Current clinical management of gastrointestinal stromal tumor. World J Gastroenterol.

[6] Kelly CM, Gutierrez Sainz L, Chi P (2021). The management of metastatic GIST: current standard and investigational therapeutics. J Hematol Oncol.

[7] Miettinen M, Lasota J (2006). Gastrointestinal stromal tumors: review on morphology, molecular pathology, prognosis, and differential diagnosis. Arch Pathol Lab Med.

[8] Deshaies I, Cherenfant J, Gusani NJ (2010). Gastrointestinal stromal tumor (GIST) recurrence following surgery: review of the clinical utility of imatinib treatment. Ther Clin Risk Manag.

[9] Nilsson B, Bümming P, Meis-Kindblom JM (2005). Gastrointestinal stromal tumors: the incidence, prevalence, clinical course, and prognostication in the preimatinib mesylate era--a population-based study in western Sweden. Cancer.

[10] Janeway KA, Kim SY, Lodish M (2011). Defects in succinate dehydrogenase in gastrointestinal stromal tumors lacking KIT and PDGFRA mutations. Proc Natl Acad Sci U S A.

[11] Gerrish ST, Smith JW (2008). Gastrointestinal stromal tumors—diagnosis and management: a brief review. Ochsner J.

[12] Parab TM, DeRogatis MJ, Boaz AM (2019). Gastrointestinal stromal tumors: a comprehensive review. J Gastrointest Oncol.

[13] Mearadji A, den Bakker MA, van Geel AN (2008). Decrease of CD117 expression as possible prognostic marker for recurrence in the resected specimen after imatinib treatment in patients with initially unresectable gastrointestinal stromal tumors: a clinicopathological analysis. Anticancer Drugs.

[14] Hirota S, Isozaki K, Moriyama Y (1998). Gain-of-function mutations of c-kit in human gastrointestinal stromal tumors. Science.

[15] Heinrich MC, Corless CL, Duensing A (2003). PDGFRA activating mutations in gastrointestinal stromal tumors. Science.

